# Infectious Salmon Anaemia Virus (ISAV) RNA Binding Protein Encoded by Segment 8 ORF2 and Its Interaction with ISAV and Intracellular Proteins

**DOI:** 10.3390/v8020052

**Published:** 2016-02-18

**Authors:** Christel M. Olsen, Turhan Markussen, Bernd Thiede, Espen Rimstad

**Affiliations:** 1 Faculty of Veterinary Medicine and Biosciences, Department of Food Safety & Infection Biology, Norwegian University of Life Sciences (NMBU), 0454 Oslo, Norway; christel.olsen@gmail.com (C.M.O.); turhan.markussen@nmbu.no (T.M.); 2 Department of Biosciences, P.O. Box 1066 Blindern, University of Oslo, 0316 Oslo, Norway; bernd.thiede@ibv.uio.no

**Keywords:** infectious salmon anaemia virus, ISAV, orthomyxovirus, gene segment 8, biscistronic, ISG15, ubiquitin, LC-MS

## Abstract

Infectious salmon anaemia virus (ISAV) is an orthomyxovirus infecting salmonid fish. The virus is adapted to low temperature and has a replication optimum between 10–15 °C. In this study the subcellular localization and protein interactions for the protein encoded by the largest open reading frame of gene segment 8 (s8ORF2) were investigated. In ISAV infected cells the s8ORF2 protein was found mainly in the cytosol but a minor fraction of cells expressed the protein in the nucleus as well. Green fluorescent protein-tagged s8ORF2 did not leak out of the cell when the plasma membrane was permeabilized, suggesting interactions with intracellular structural components. The s8ORF2 protein exists both as monomer and homodimer, and co-immunoprecipitation experiments strongly suggests it binds to the ISAV fusion-, nucleo- and matrix proteins. Two versions of s8ORF2 were detected with apparent molecular weights of 24–26 and 35 kDa in lysates of infected cells. The 35 kDa type is an early viral protein while the smaller version appears during the later phases of infection. The 24–26 kDa type was also the predominant form in viral particles. The s8ORF2 protein has previously been shown to bind RNA and interfere with interferon induction and signaling. Here we found that a fraction of the s8ORF2 protein pool in infected cells is likely to be conjugated to the interferon stimulated gene 15 (ISG15) and ubiquitin. Furthermore, several endogenous proteins pulled down by the s8ORF2 protein were identified by liquid chromatography mass spectrometry (LC-MS).

## 1. Introduction

Infectious salmon anaemia virus (ISAV) belongs to the genus Isavirus in the family *Orthomyxoviridae*. The virus has a genome of eight single-stranded, negative-sense RNA segments that encode at least 10 proteins [1,2,3]. It is an important pathogen of farmed Atlantic salmon (*Salmo salar*) and the cumulative mortality can exceed 90% during an outbreak. In order to control the spread of the disease, mandatory depopulation of net pens and other biosecurity measures managed by the Norwegian Food Safety Authority have been introduced in recent years. Similar to influenza viruses (*Orthomyxoviridae*) the two smallest gene segments of ISAV encode more than one protein. ISAV segment 8 has two overlapping collinear open reading frames (ORFs). The smallest ORF encodes the matrix (M) protein [2,4] and the larger (s8ORF2) encodes a protein that binds both single- and double-stranded RNA and antagonizes the type I interferon (IFN) response [5,6]. The corresponding gene segment in influenza A viruses (IAV), segment 7, encode the matrix (M1) and the ion channel protein (M2) from collinear and spliced mRNA, respectively. No homologue to the influenza virus ion channel protein has been identified in ISAV.

Infection with ISAV leads to activation and production of type I interferon (IFN) in Atlantic salmon and in cell lines derived from Atlantic salmon [7,8,9]. The type I IFNs are produced in response to viral nucleic acids detected by pattern recognition receptors (PRRs) and lead to transcription of interferon stimulated genes (ISGs) [10]. ISG15 is an ubiquitin-like protein modifier that is strongly induced by type I IFN (review in [11]), and it targets viral proteins from various virus families. The antiviral mechanisms at work can be diverse and executed by both conjugated and unconjugated forms of ISG15 [11]. ISG15 and other protein post-translational modifiers such as ubiquitin (Ub) and small ubiquitin-like modifier (SUMO) are central in fine-tuning antiviral innate immune responses and participate in regulating activity, stability, affinity and location of many signalling proteins (review in [12]). However, there is no evidence that ISGylation results in the proteasome-mediated degradation of target proteins [11]. NS1, the type I IFN-antagonizing protein from IAV and influenza B virus (IBV) are both ISGylated [13,14]. ISG15 modification of NS1 inhibits IAV replication and thus contributes to the antiviral action of type I IFN. However, ISG15 and dsRNA can bind simultaneously and the binding of the ISG15 protein does not seem to have detectable effect on the dsRNA binding of the NS1. NS1-IBV only binds ISG15 molecules of human and non-human primate origin and could be a host-restricting factor for this virus, which is known to cause disease only in humans [15,16]. However, IBV has also occasionally been detected in seals [17,18,19]. For ISAV, previous studies have indicated that a low-virulent strain induced the antiviral proteins Mx and ISG15 more potently than a high virulent ISAV strain early in the infection cycle in cell cultures [20]. Similarly, infection with a low-virulent ISAV strain produced higher levels of type I IFN and Mx earlier than a high virulent strain in an experimental infection trial [21].

In this study the sub-cellular localisation of the ISAV s8ORF2 protein was studied and shown to be predominantly cytosolic. The protein is present both as a monomer and a dimer and was found in two forms; an early variant with higher molecular weight (MW) than predicted and a smaller variant that is more prominent in the virus particle. A fraction of s8ORF2 protein is conjugated to ISG15 while a minor fraction is conjugated to ubiquitin. In co-immunoprecipitation experiments, s8ORF2 protein binds to the ISAV fusion- (F), nucleo- (NP) and matrix- (M) proteins. LC-MS analysis indicates that s8ORF2 interacts with proteins associated with the cytoskeleton, ribosomal proteins, mRNA processing proteins and voltage-dependent anion channels.

## 2. Materials and Methods 

### 2.1. Cells, Virus, Cloning and Transfections

Atlantic salmon head kidney cells, ASK-cells, [22] and EPC cells (ATCC CRL-2872, Epithelioma papulosum cyprinid) were grown in Leibovitz L-15 media (Gibco) added gentamycin (50 µg/mL), 2-mercaptoethanol (40 µM) and 10% foetal bovine serum (PAA). The ISAV strain Glesvaer/2/90 [23] passaged four times in SHK-1 cells and two times on ASK cells was used in the experiments. Aliquots of the same virus batch were used for all experiments, and s8ORF2 was sequenced (GATC-Biotech AG, Konstanz, Germany). The tissue culture infective dose (TCID_50_/mL) of ISAV were found to be 5 × 10^6^/25 µL using Kärbers formula after immunostaining with anti-ISAV antibody (Aquatic Diagnostics, Stirling, UK) five days post infection (dpi). Viral particles were purified from supernatant of infected ASK cells eleven dpi. In short, cell debris was removed by centrifugation at 6000× *g* for 25 min (Sorvall RC-5B, F15-8 × 50c) followed by ultracentrifugation at 111,000× *g* for 1.5 h (Beckman, SW40Ti). Pellet containing viral particles was dissolved in PBS. Supernatant from non-infected cells undergoing the same procedure was used as negative control. EPC cells were used for transfection experiments. Four million cells were transfected with 4 µg pcDNA3.1-s8ORF2 by electroporation using the AMAXA™ Nucleofector™ (Lonza, Basel, Switzerland) and Ingenio transfection reagents (Mirus Bio, Madison, WI, USA). This construct was made by cloning the Glesvaer s8ORF2 gene (Genbank acc. no. DQ785276) into the EcoRI/XhoI restriction sites of the pcDNA3.1+Myc-His vector C (Invitrogen Thermo-Fisher, Waltham, MA, USA). Plasmids were purified using the QIAprep Spin Miniprep Kit (Qiagen, Hilden, Germany) and the inserts were sequenced (GATC-Biotech AG, Konstanz, Germany).

#### 2.1.1. Immunostaining and Microscopy

ASK cells (5 × 10^4^/well) were seeded on glass-coverslips (Assistant) in a 24 well-plate, infected with ISAV after 24 h (250 µL at 1:3 dilution) and added fresh media 3 h post infection (hpi). Coverslips were fixed in 80% acetone at 6, 16, 24, 27, 30, 48 and 72 hpi, rehydrated in PBS, blocked with 5% skimmed milk in PBS and stained with rabbit-anti-s8ORF2 (1:1000, [5]) or mouse-anti-ISAV (Aquatic diagnostics, 1:500) in 1% skimmed milk in PBS. Anti-rabbit Alexa-Fluor-488 and anti-mouse Alexa-Fluor-594 were used as secondary antibodies (Molecular Probes). Coverslips were mounted with Fluoroshield (Sigma) and microscopy performed using an Olympus IX81 microscope. To investigate CRM1–dependent nuclear export, ASK cells were treated with 40 nM Leptomycin B (Sigma-Aldrich) in the media for 4 h prior to immunostaining. Non-saturated images were captured by a Plan-Apochromate 63×/1.4 oil objective in a Zeiss laser scanning confocal microscope (Zeiss Axiovert 200M fluorescent inverted microscope, equipped with a LSM 510 laser confocal unit and 488 nm argon laser and 546 nm helium/neon laser, Carl Zeiss, Jena, Germany). For live-imaging, EPC cells were transfected with p8ORF2-EGFP [5] or pEGFP-N1 (Clontech Laboratories). A total of 4 × 10^5^ cells/well were seeded in eight-well chamber slides (Lab-Tec, Nunc, Thermo-Fisher, Waltham, MA, USA) coated with 1% gelatin. 24 h post transfection (hpt), cells were washed with KHM-buffer (100 mM potassium acetate, 20 mM HEPES, pH 7.2, 2 mM MgCl_2_). Images were captured in an Olympus IX81 inverted fluorescence microscope at fixed intervals following addition of 160 µM digitonin (Sigma, Aldrich St-Louis, MO, USA) to the KHM-buffer for selective permeabilization of the plasma membrane.

#### 2.1.2. Immunoprecipitation (IP) and SDS-PAGE

ASK-cells were seeded to 80%–90% confluence in T-162 flasks and the following day infected with 4 mL of ISAV (1:5 dilution). Virus inoculate was replaced after 3 h with fresh media containing 2% FCS. Cells were harvested 3 dpi using a rubber policeman and lysed in RIPA buffer (50 mM Tris-HCl, pH 7.5, 150 mM NaCl, 2 mM EDTA and 1% NP-40) at 4 °C for 30 min, followed by centrifugation at 1150× *g* for 5 min. Post-nuclear supernatants were immunoprecipitated overnight with polyclonal antibodies against ISAV s8ORF2 [5] or matrix (M) [4], ISAV fusion protein (F) [24] or monoclonal antibody against NP (anti-ISAV, Aquatic Diagnostics). The following day pull-down was performed using the Dynabeads Protein G immunoprecipitation kit (Novex, Thermo-Fisher, Waltham, MA, USA ) following the protocol by the manufacturer. For SDS-PAGE, magnetic bead pellets were boiled for 10 min in NuPAGE loading buffer with reducing agent (BioRad, Hercules, CA, USA). Samples without reducing agent were heated at 60 °C for 10 min. Non-solvable particles were pelleted by centrifugation at 10,000× *g* for 5 min. Proteins were separated on a 4%–12% linear gradient Bis-Tris PreCast gel Criterion™ Cell (BioRad) with XT-MOPS as running buffer. Following blotting onto polyvinylidene difluoride (PVDF) membranes (BioRad), the proteins were detected with anti-s8ORF2 antibody, anti-salmon ISG15 antibody [25] or anti-ubiquitin antibody (P4D1, Enzo). Anti-hemagglutinin-esterase [2], anti-NEP [26] and anti-NS [5] were also tested on proteins pulled down by anti-s8ORF2 antibody. Secondary antibodies were anti-rabbit IgG HRP conjugated (GE Healthcare, Little Chalfont, UK) or Rabbit-Trueblot® (eBioscience, San Diego, CA, USA). Proteins were visualized using Amersham™ ECL Prime Western Blotting detection reagent (GE Healthcare). For time-laps experiments, 10^6^ ASK-cells in T-25 cm^2^ flasks were infected 24 h after seeding with 1 mL 1:5 dilution of ISAV. After 3 h, media was replaced with L-15 containing 2% FCS, and cells were harvested 1–4 dpi.

#### 2.1.3. Immunoprecipitation, LC-MS and Data Analysis

Post-nuclear supernatants from infected ASK-cells or transfected EPC cells were immunoprecipitated with anti-s8ORF2 antibody overnight, and pulled down by Dynabeads Protein G. The resulting bead pellets were boiled for 5 min in 30 µL NuPAGE sample buffer with reducing agent. Beads were removed by magnet and supernatants run on SDS-PAGE as described above. The gel was stained with Simply Blue Safe Stain (Invitrogen) and target bands were excised using a scalpel for in-gel digestion in 20 µL of 50 mM ammonium bicarbonate, pH 7.8 with 0.1 µg of trypsin (Promega, Madison, WI, USA). 

After micropurification using µ-C18 ZipTips (Millipore, Oslo, Norway), the peptides were dried in a SpeedVac and dissolved in 10 µL 1% formic acid, 5% acetonitrile in water. Half of the volume was injected into an Ultimate 3000 nanoLC system (Dionex, Sunnyvale, CA, USA) connected to a linear quadrupole ion trap-orbitrap (LTQ-Orbitrap XL) mass spectrometer (ThermoScientific, Bremen, Germany) equipped with a nanoelectrospray ion source. For liquid chromatography separation, an Acclaim PepMap 100 column (C18, 3 µm beads, 100 Å, 75 μm inner diameter) (Dionex, Sunnyvale, CA, USA) capillary of 50 cm bed length was used. The flow rate was 0.3 μL/min, with a solvent gradient of 7% B to 35% B in 40 min. Solvent A was aqueous 0.1% formic acid, whereas solvent B was aqueous 90% acetonitrile in 0.1% formic acid. The mass spectrometer was operated in the data-dependent mode to automatically switch between Orbitrap-MS and LTQ-MS/MS acquisition. Survey full scan MS spectra (from *m/z* 300 to 2000) were acquired in the Orbitrap with the resolution *R* = 60,000 at *m/z* 400 (after accumulation to a target of 1,000,000 charges in the LTQ). The method used allowed for the sequential isolation of up to the seven most intense ions, depending on signal intensity, for fragmentation on the linear ion trap using collision induced dissociation (CID) at a target value of 10,000 charges. Target ions already selected for MS/MS were dynamically excluded for 60 s. The lock mass option was enabled in MS mode for internal recalibration during the analysis. Other instrument parameters were set as previously described [27].

Data were acquired using Xcalibur v2.5.5 and raw files were processed to generate peak list in Mascot generic format (*.mgf) using ProteoWizard release version 3.0.331. Database searches were performed using Mascot (Matrix Science, London, UK; version 2.4.0). Mascot was set up to search the NCBInr_20131127 database (selected for Viruses, 1250796 entries or ISAV Glesvaer strain, 10 entries) and NCBInr_20131127 database (selected for Other Actinopterygii, 364677 entries) assuming the digestion enzyme trypsin. Mascot was searched with a fragment ion mass tolerance of 0.60 Da and a parent ion tolerance of 10 PPM. Oxidation of methionine, acetyl of the n-terminus and propionamide of cysteine were specified in Mascot as variable modifications. In addition, database searches were performed considering phosphorylation of serine, threonine and tyrosine and modifications of lysines with Gly-Gly and Leu-Arg-Gly-Gly to find phosphorylation and ubiquitinylation sites, respectively. Scaffold (version Scaffold_4.3.4, Proteome Software Inc., Portland, OR) was used to validate MS/MS based peptide and protein identifications. Peptide identifications were accepted if they could be established at greater than 95.0% probability by the Scaffold Local false discovery rate (FDR) algorithm. Protein identifications were accepted if they could be established at greater than 99.0% probability and contained at least 2 identified peptides.

#### 2.1.4. Computational Analysis

Theoretical molecular weights for proteins were calculated using the Compute pI/Mw tool [28]. SignalP [29] and TMpred [30] were used for prediction of signal peptide sequences and transmembrane helixes, respectively. The presence of putative N-glycosylation sites in the s8ORF2 protein was investigated using NetNGlyc 1.0 [31] and PSIPRED v3.3 was used to predict protein secondary structures [32]. The degree of hydrophobicity of the s8ORF2 protein was investigated using ProtScale [33] and the algorithm by Kyte and Doolittle [34], and the GRAVY score was calculated using the Gravy Calculator [35]. The presence of sequences rich in proline (P), glutamic acid (E), serine (S) and threonine (T) (putative PEST sequences) in ISAV proteins was investigated using epestfind [36]. NoD (Nucleolar localization sequence Detector) was used to search for putative nucleolar localization sequences (NoLSs) in the s8ORF2 protein [37].

## 3. Results

### 3.1. The s8ORF2 Protein Exists in Different Forms in Infected Cells

**Figure 1:**
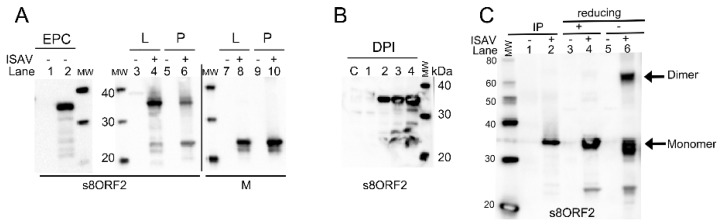
Western blots of lysates from EPC cells transfected with the s8ORF2 construct, ISAV infected ASK cells, and viral particles. (**A**) EPC-cells; lane 1: non-transfected, lane 2: transfected with s8ORF2 construct. Lanes 3–10: ASK-cell lysates (L) and virus particles (P) of non-infected (−, lanes 3, 5, 7 and 9) and 4 days post infection (dpi) (+, lane 4, 6, 8, 10). Lanes 1-6 display staining with anti-s8ORF2 antibody and 7–10 with anti-M antibody; (**B**) ASK cells infected with ISAV and harvested 1–4 dpi and control lysate from non-infected cells (**C**); (**C**) ASK cell lysates immunoprecipitated with anti-s8ORF2 antibody. Lane 1: control and lane 2 = 4 dpi. Lanes 4 and 6: non-immunopreciptated lysates under reducing and non-reducing conditions, respectively. Lanes 3 and 5: control lysates from non-infected cells under corresponding conditions. MW = molecular weight standard.

Expression of s8ORF2 in transfected EPC cells (cyprinid origin) produced a protein of approximately 35 kDa (Figure 1A, lane 2). No signal was seen in lysates from non-transfected cells (Figure 1A, lane 1). Interestingly, in lysates from ISAV infected ASK cells (salmonid origin), additional bands representing protein sizes of approximately 25, 27 and 32 kDa were observed (Figure 1A, lane 4). The 27 kDa band was also observed from purified viral particles in addition to the 35 kDa band (Figure 1A, lane 6). The predicted size of the s8ORF2 protein is 27.4 kDa. SignalP and TMpred do not predict the presence of a signal peptide or transmembrane regions, respectively. Two N-glycosylation sites, at positions 36 and 144, are predicted for this protein although these are not likely to be functional in the absence of a signal peptide sequence. 

The protein representing the 35 kDa band was heavily stained in lysates from both transfected EPC cells and infected ASK cells, but dominated less in viral particles. Comparable amounts of viral proteins from cell lysate and virus pellet were loaded onto the gel as indicated by similar band signal intensities when the 22 kDa ISAV Matrix (M) protein was targeted (Figure 1A, lanes 8 and 10). A time course infection study showed that the 35 kDa s8ORF2 band was weak on day 1 pi but increased significantly in intensity on day 2. In contrast, the two smaller bands were weak at 2 and 3 dpi but displayed significantly stronger staining 4 dpi (Figure 1B). Interestingly, when the protein representing the 35 kDa band was subjected to LC-MS analysis, only peptides corresponding to the s8ORF2 protein sequence were detected. In ISAV infected cells, s8ORF2 was also present as dimers as indicated by the band representing protein(s) of approximately 65 kDa when non-reducing conditions were applied in western blot (Figure 1C, lane 6). When anti-s8ORF2 was used in immunoprecipitation it displayed strongest affinity for the 35 kDa form of the protein (Figure 1C, lane 2). 

#### 3.1.1. The s8ORF2 Protein Localizes Mainly to the Cytoplasm but is also Present in the Nucleus in ASK Cells 

**Figure 2:**
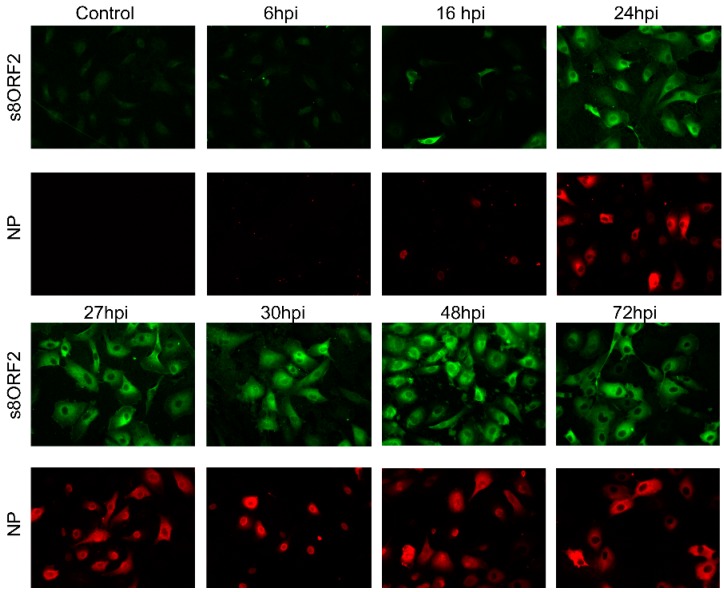
Subcellular localization of the s8ORF2 protein (green) and NP (red) in ISAV infected ASK cells. ISAV infected cells were immunostained separately with anti-s8ORF2 or anti-ISAV (NP) at 6, 16, 24, 27, 30, 48 and 72 h post infection (hpi). Controls are non-infected cells. Images were captured by fluorescence microscopy at 20× magnification.

**Figure 3:**
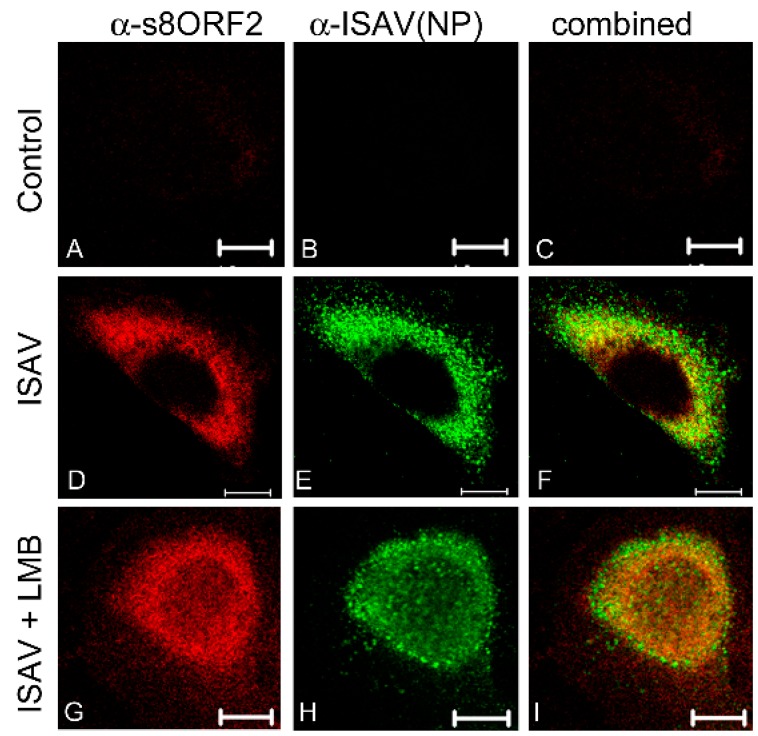
Subcellular localization of the s8ORF2 protein following treatment with the CRM-1 dependent nuclear export inhibitor leptomycin B (LMB). The s8ORF2 protein displays both cytosolic and nuclear localization and was retained in the nucleus following addition of leptomycin B (LMB). ISAV infected ASK cells were 48 h post infection exposed to 40 µM LMB for 4 hours followed by immunostaining with anti-s8ORF2 antibody (red) and anti-NP antibody (green). (**A**–**C**) Non-infected control cells; (**D**–**F**) ISAV infected cells; (**G**–**I**) ISAV infected and LMB exposed. Images were captured by confocal microscopy at 63x magnification. Combined signals = yellow. Scale bar = 10 µm.

A time course study was performed where ISAV infected ASK cells were immunostained 6–72 hpi (Figure 2). The earliest time point s8ORF2 protein could be detected was 16 hpi, similar to that of the early viral protein NP. The s8ORF2 protein localized primarily to the cytoplasm, although nuclear staining could be observed in some cells during this time period. Z-stacking in confocal scanning microscope of ISAV infected ASK cells 72 hpi confirmed that the s8ORF2 protein at some stages during the infection cycle displayed both cytoplasmic and nuclear localization (S1). Furthermore, when CRM-1 dependent nuclear export was inhibited using leptomycin B on infected cells, both NP and s8ORF2 protein displayed nuclear retention (Figure 3 G–I), but this was not observed when s8ORF2 was over-expressed alone (S2).

#### 3.1.2. The s8ORF2 Protein is Bound to Intracellular Structures Preventing it from Diffusing Freely out of the Cytoplasm Following Plasma Membrane Permeabilization 

**Figure 4:**
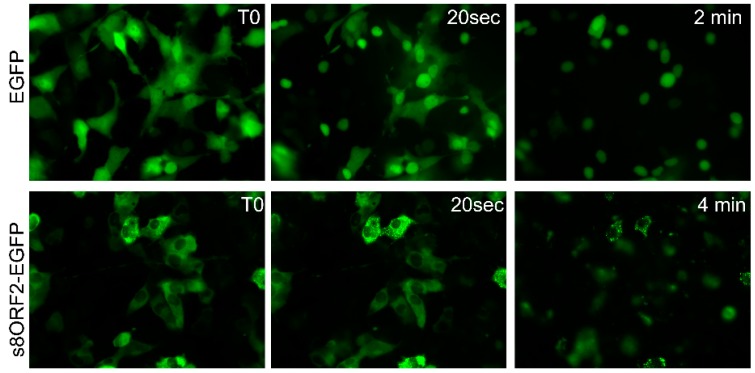
Live images of EPC cells transfected with constructs expressing EGFP or s8ORF2-EGFP captured with time-laps microscopy after permeabilization of cell membranes with digitonin. Cells were treated with a hypotonic buffer added 160 µM digitonin and images were captured at fixed intervals at 40× magnification in an inverted fluorescence microscope. T0 = time zero.

EPC cells transfected with EGFP displayed fluorescent signal in both nucleus and the cytosol. In contrast, the signal from s8ORF2-EGFP was predominantly cytosolic (Figure 4 T0). Following addition of digitonin, which permeabilizes the plasma membrane, EGFP diffusion out of the cytoplasm was apparent after 20 s with no visible cytoplasmic staining remaining in most cells after 2 min. In contrast, s8ORF2-EGFP did not diffuse rapidly out of the cell following permeabilisation, as only minimal change in fluorescent signal was observed after 20 s. After 4 min weak fluorescent signals were observed in most s8ORF2-EGFP transfected cells. A few cells did though display strong fluorescence in the form of granular patterns, suggesting that the s8ORF2-EGFP protein may interact with intracellular structures that prevent it from diffusing freely out of the cell.

#### 3.1.3. The s8ORF2 Protein Interacts with the ISAV Nucleo-, Matrix and Fusion Proteins

**Figure 5:**
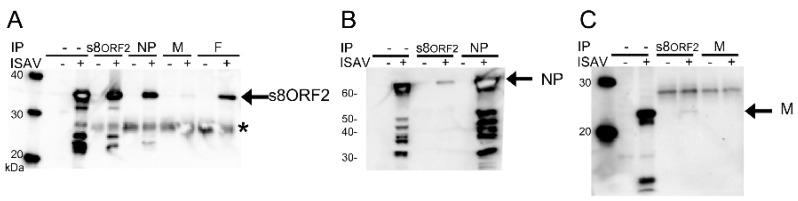
Western blots of co-immunoprecipitation (IP) assays with s8ORF2 and ISAV proteins NP, M and F. Four days post infection of ASK cells, IP shows that s8ORF2 protein interacts with the ISAV nucleoprotein (NP), matrix protein (M) and fusion protein (F). Cell lysates from non-infected (−) or ISAV infected cells (+) were IP with either anti-s8ORF2 antiserum, anti-NP mAb, anti-M- or anti-F antiserum, or run directly on western blots (WB) without IP (−). (**A**) WB with anti-s8ORF2 antiserum; (**B**) WB with anti-NP antibody; (**C**) WB with anti-M antiserum. Arrows mark bands representing full-length proteins. Additional bands might represent cleavage products except (*) that is unspecific signal.

Immunoprecipitation indicated that the s8ORF2 protein interacts with other ISAV proteins; the nucleoprotein (NP), matrix protein (M) and fusion protein (F) in cell lysates from infected cells (Figure 5A–C). Anti-s8ORF2 antiserum used in western blotting detected the 35 kDa band representing the s8ORF2 protein after pull-down with anti-NP mAB, anti-M- or anti-F antiserum (Figure 5A). When the opposite was assayed, *i.e.*, pull-down with anti-s8ORF2 and WB with anti-NP- or anti-M antiserum, bands corresponding to the expected sizes of NP (68 kDa) and M (22 kDa) was observed (Figure 5B,C). For the F protein, however, an unspecific protein band with approximately the same MW as the F protein co-precipitated with anti-s8ORF2 making these results difficult to interpret (S3). The staining of M after using anti-M in immunoprecipitation was very weak indicating that this antibody has low affinity to the native protein (Figure 5C). Also, a comparatively low amount of M was pulled down using the anti-s8ORF2 in immunoprecipitation (Figure 5C). A heavily stained band below 20 kDa from a non-IP sample was also observed in the M-blot (Figure 5C), possibly indicating a cleavage product of the M protein. Similar immunoprecipitation assays with the s8ORF2 protein targeting the ISAV proteins haemagglutinin-esterase (HE), the non-structural (NS) protein and the nuclear exporting protein (NEP) provided no bands in WB of expected protein sizes, which strongly suggests that the s8ORF2 protein does not interact with these three proteins. However, only the s8ORF2 protein and NP were identified in peptide analyses of visible protein bands after pull-down using anti-s8ORF2 antiserum (Table 1). 

#### 3.1.4. In Infected Cells the s8ORF2 Protein Is Conjugated to ISG15 and Ubiquitin

**Figure 6:**
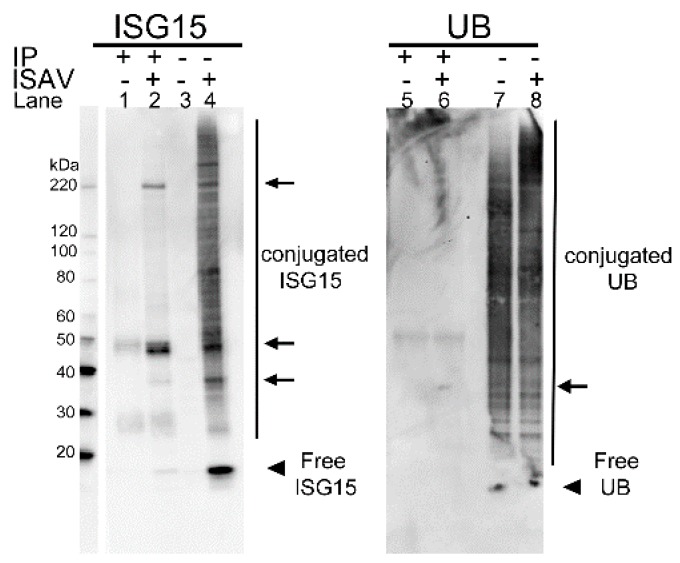
Western blots of co-immunoprecipitation (IP) assays using anti-s8ORF2 antiserum and targeting ISG15 and ubiquitin. Interferon stimulated gene 15 (ISG15) and ubiquitin (UB) conjugated to s8ORF2 protein were pulled down by anti-s8ORF2 antiserum from ISAV infected ASK cells. Lanes 1–4: staining with anti-ISG15 antiserum; Lanes 5–8: staining with anti-UB antibody, Lanes 3, 4, 7 and 8: non-IP cell lysates; Lanes 1, 2, 5 and 6: IP proteins. Lanes 1, 3, 5 and 7: Non-infected controls (−). Lanes 2, 4, 6 and 8 four days post infection with ISAV (+). ISG15- and UB-conjugated s8ORF2 protein bands are indicated by arrows, and free ISG15 and UB by arrowheads.

The interferon stimulated gene 15 (ISG15) was strongly induced during ISAV infection as observed by a smear of ISG15-conjugated proteins with MWs from 25 kDa and upwards in lysates from infected cells compared to non-infected (Figure 6, lane 4 and 3, respectively). Immunoprecipitation using anti-s8ORF2 antiserum revealed in WB bands of ISG15-conjugated proteins displaying approximate sizes of 39 kDa, 49 kDa and one slightly above 220 kDa (Figure 6, lane 2). The s8ORF2 protein was also detected by LC-MS sequencing of the ~49 kDa protein band (Table 1). When staining for ubiquitin, a weak band of approximately 35 kDa was observed indicating that a minor fraction of s8ORF2 protein is ubiquitinylated (Figure 6, lane 6). However, ubiquitin was not induced by the infection as observed with ISG15 (*i.e.*, Figure 6, lanes 7, 8 *vs.* lane 3, 4, respectively). 

**Figure 7:**
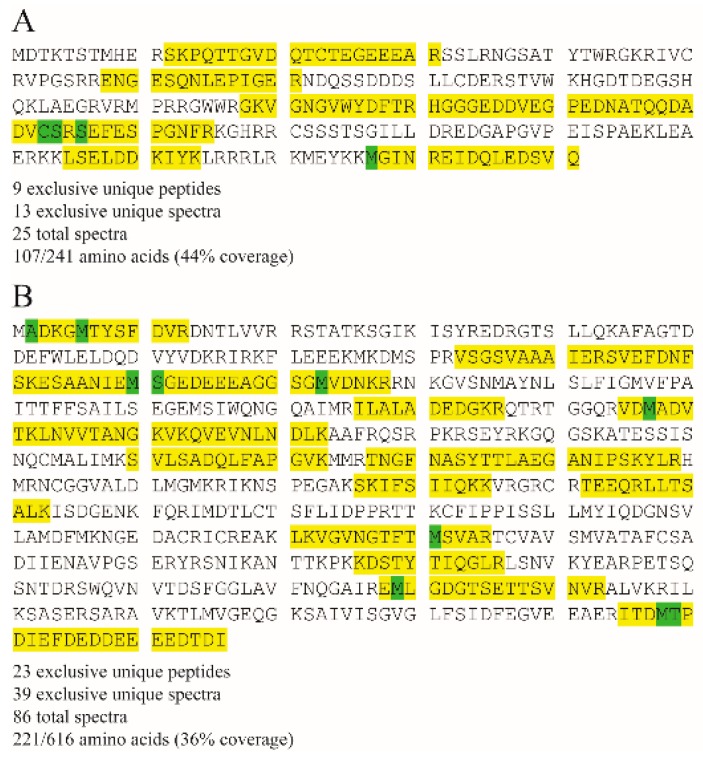
Identified peptides are highlighted in yellow, modifications are highlighted in green (cysteine propionamidation (+71 Da), methionine oxidation (+16 Da), N-terminal acetylation (+42 Da), and phosphorylation (+80 Da)). Modification of cysteines with propionamide and oxidation of methionine are well-known artificial modifications. (**A**) Phosphorylation of serine-154 in the s8ORF2 protein within the peptide HGGGEDDVEGPEDNATQQDADV**CS**R**S**EFESPGNFR was identified, whereas phosphorylation of serine-156 could not unequivocally be verified by manual inspection of the corresponding MS/MS spectrum; (**B**) The NP protein was modified at alanine-2 (green) by removing the N-terminal methionine and acetylation of the following amino acid (**A**DKG**M**TYSFDVR). Phosphorylation of serine-111 could be confirmed by identification of ESAANIE**MS**GEDEEEAGGSG**M**VDNK and SVEFDNFSKESAANIE**MS**GEDEEEAGGSG**M**VDNK. Phosphorylation of threonine-599 was found within the peptide ITD**MT**PDIEFDEDDEEEEDTDI.

In bioinformatic search for ubiquitination signals, using default settings, epestfind predicted two putative PEST sequences in the s8ORF2 protein, in positions 14–30 and 132–154, the former with the highest score. PEST sequences are rich in proline (P), glutamic acid (E), serine (S) and threonine (T) which can be linked to ubiquitinylation of proteins [38]. Also, phosphorylation of serines or threonines may activate latent PEST sequences. The N-terminal predicted PEST sequence in the s8ORF2 protein contains four threonines and no serines, while the second motif contains one threonine. A search for modifications in the identified s8ORF2 and NP peptides from LC/MS analysis identified putative phosphorylations of serine-154 and possibly serine-156 in the s8ORF2 protein (Figure 7). Position 154 lies within one of the two PEST motifs predicted to be present in this protein (Figure 7A). However, in the Glesvær strain, the amino acid in position 154 in the s8ORF2 protein is a proline. To investigate this discrepancy, s8ORF2 from the virus batch used to infect the cells that were analyzed by MS was re-sequenced. The re-sequencing confirmed this position to be occupied by a proline, similar to the cloned s8ORF2 construct. Serine-156, on the other hand, lies two positions downstream of the predicted PEST sequence. Of the NP peptides, three modifications were identified; acetylation of alanine-2 and phosphorylation of serine-111 and threonine-599 (Figure 7B). It cannot be excluded that threonine/serine phosphorylation is involved in regulating functional properties of the s8ORF2 protein. Such functions may include cellular localization, protein activation/inactivation of proteins, protein-protein interactions or degradation [38,39,40,41]. No PEST sequences could be predicted in any of the remaining known eleven ISAV proteins (not shown). 

#### 3.1.5. Proteins Associated with Cytoskeleton, Ribosomes, Mitochondria, and Cell Membranes Were Identified in Peptide Analysis Following Immunoprecipitation with Anti-s8ORF2

**Figure 8:**
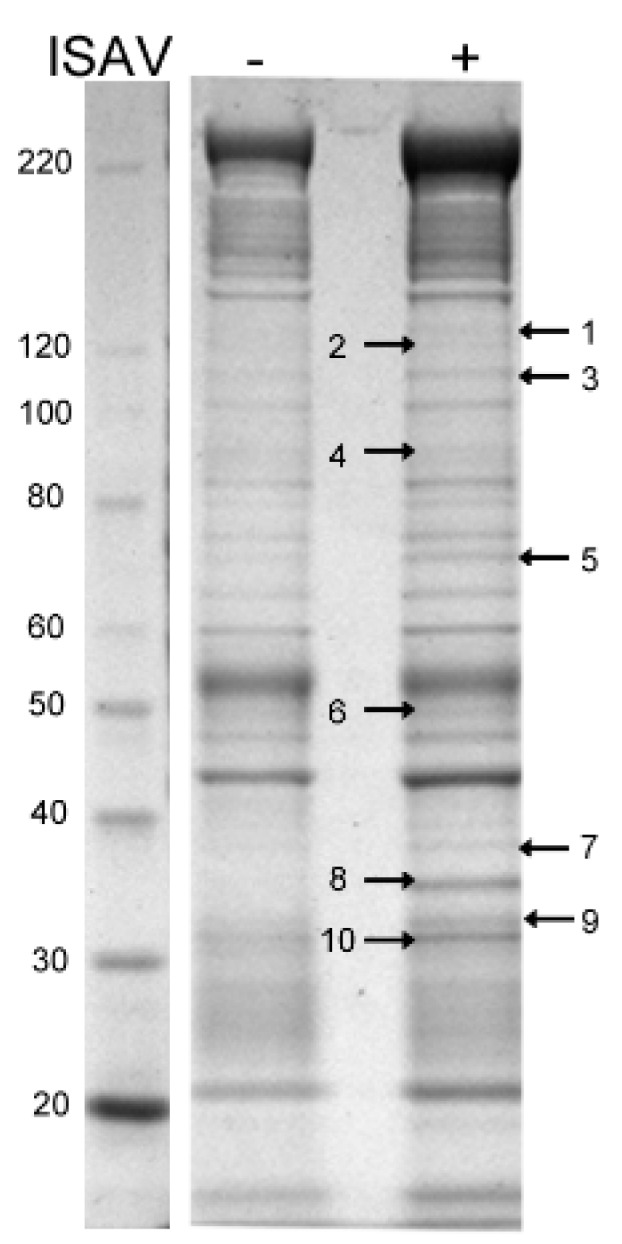
Commassie stained protein gel of ASK cell lysates and proteins pulled down by immunoprecipitation with anti-s8ORF2 from non-infected (−) and ISAV infected (+) cells four days post infection. Arrows and numbers indicate protein bands excised for LC/MS analysis.

LC-MS analysis of the visual protein bands pulled down by anti-s8ORF2 from virus infected cells revealed proteins of the cytoskeleton, proteins involved in protein folding and RNA binding, mitochondrial membrane proteins and proteins involved in vesicle transport (Figure 8, Table 1).

Of the cytoskeletal proteins pulled down, the majority were associated with actin-filaments and a few with intermediate filaments and microtubules. Chaperones, such as heat shock protein 90 (HSP90) and heat shock cognate 71 protein (HSC71), and mitochondrial membrane proteins like the voltage dependent anion channel and inner membrane protein, were also identified. Annexins and flotellins, proteins involved in endo-and exocytosis as well as several ribosomal proteins and proteins involved in mRNA transport, were identified as well. The ISAV NP and s8ORF2 proteins were also present in several of the protein bands excised (Table 1). However, neither ISAV F or M, ISG15 nor Ubiquitin were identified from any of the protein bands analyzed. 

## 4. Discussion

The ISAV s8ORF2 protein seems to exist in at least two forms as previously shown by Garcia-Rosado *et al.* [5]. In this study, we found that the 35 kDa variant was expressed early during the infection cycle and to be the dominant form in lysates from infected cells. Smaller protein variants began to appear three days post infection coinciding approximately with the time of viral budding from the plasma membrane. As assembled viral particles are not found inside the infected cell, this could explain why the smaller variant of the protein was the dominant form in the virus pellet. The estimated molecular weight of the band representing the smaller protein variant, 24–25 kDa, was slightly less than that predicted for the s8ORF2 protein (27.4 kDa), and the size estimated in a previous study (27 kDa) [5]. As the Glesvær strain was used in both studies, the size differences observed in the former and latter experiments may be explained by the use of different MW markers producing slightly different travel distances in the gel. A GRAVY score of −1.3 and ProtScale analyses (not shown) places the s8ORF2 protein on the hydrophilic side (not shown).

Interestingly, when the s8ORF2 protein was expressed following transfection, only the 35 kDa form of the protein was observed. Hence, other ISAV proteins are not involved in the observed weight discrepancy, suggesting these proteins or infection-induced signals are necessary for producing the smaller variant(s). Furthermore, only peptides present in s8ORF2 protein were found when the 35 kDa protein was sequenced by MS. 

The protein band(s) corresponding to the 27 kDa variant did not appear until 3 dpi. Hence, it is the 35 kDa version of the protein that is stained in infected cells until 3 dpi. As this variant was observed as early as 16 hpi, it might be an early and modified form of this viral protein, containing a modification that is removed during viral particle assembly, which could explain why the smaller variant was not observed during over-expression. However, in contrast to the previous study by Garcia-Rosado and co-workers [5] we found the s8ORF2 protein to have a predominantly cytosolic localization in both infected cells and in cells expressing the larger s8ORF2-EGFP variant (EGFP = 240 aa). Only a few infected cells expressed s8ORF2 protein both in the nucleus and cytosol 30 hpi and onwards. This might indicate that s8ORF2 protein locates to the nucleus transiently as cells in the previous study were stained 18 hpi [5]. Two putative nuclear localization signals (NLSs) have previously been predicted with PSORTII [5]. Still, the small size of the monomer should enable it to move freely between the nuclear- and cytosolic compartments. Treatment of cells with leptomycin, an inhibitor of CRM-1 dependent nuclear export, did not result in nuclear accumulation of s8ORF2 protein when the protein was expressed alone following transfection. In infected cells, however, s8ORF2 protein displayed nuclear retention suggesting that when interacting with other ISAV proteins, or cellular proteins induced by the infection, at least a fraction of s8ORF2 protein is dependent upon CRM1 assisted/mediated export from the nucleus. For IAV NS1 it has been indicated that nuclear and nucleolar localization and behavior of this protein is dependent on virus subtype [42]. For the s8ORF2 protein, NoD did not predict any NoLS (not shown), and previous studies using NetNES1.1 failed to predict putative leucine rich nuclear export signals (NESs) [5]. 

NS1 proteins from IAV, IBV and ICV have all been shown to exhibit type I IFN antagonistic properties (reviewed in [43]). Previous studies have shown that ISAV encode two proteins that inhibits type I IFN signalling, the s7ORF1 and s8ORF2 protein [5]. There are no apparent sequence identities or homologies in secondary structure between the ISAV s8ORF2 and s7ORF1 proteins, and the influenza virus’ NS1A, NS1B or NS1C as determined from multiple sequence alignments and PSIPRED, respectively (not shown). This also includes functionally important amino acids conserved in some IAV NS1 strains (reviewed in [43]). In addition, the IAV homologue is non-structural while the ISAV s8ORF2 protein is present also in the viral particle [5].

Still, similar to IAV NS1 [44], s8ORF2 protein binds RNA and probably forms homodimers. A coiled-coil domain has been predicted for the 47 C-terminal amino acids [5], and such domains are known to be involved in oligomerization [45]. In SDS-PAGE without reducing agent both the 35–40 kDa protein band and an additional band of 65–70 kDa, a size that could represent the homodimeric form of s8ORF2 protein, were found. 

The early expression of this protein led us to investigate whether the IFN-stimulated gene product ISG15 is recruited by the s8ORF2 protein. Proteomic studies have identified hundreds of proteins that are ISGylated or interact with ISG15 after IFN stimulation [14,46], and the anti-viral protein ISG15 is strongly induced during ISAV infection [8,20,25]. A smear of different ISG15-conjugated proteins was observed during ISAV infection as previously shown [20,25]. However, when s8ORF2 protein was pulled down by anti-s8ORF2, two strong and one weak band representing ISGylated s8ORF2 protein with different MWs were observed, indicating that the s8ORF2 protein is covalently linked to one or more ISG15 units. We were, however, not able to detect ISG15 by LC-MS in the 49 kDa protein band targeted for LC-MS analysis, and the MWs of the two other ISGylated s8ORF2 proteins did not match the visual protein bands targeted for LC-MS analysis. A possible explanation for the negative result in LC-MS could be that the extreme sensitivity of anti-ISG15 antiserum exceeded the sensitivity of the LC-MS. ISG15 is linked to newly synthesized proteins in an enzymatic cascade reaction involving three enzymes which are also induced by type I IFNs. In IAV infected cells, NS1 is the major viral protein targeted by ISG15, where, a lysine in position 41 (K41) has been experimentally determined to be the primary conjugation site for many IAV strains [14,47]. The ISAV s8ORF2 protein contains fifteen lysine residues, eight of which are located in the predicted 47 aa C-terminal coiled-coil region, all of which could potentially serve as acceptors for ISG15. For the IAV and IBV NS1, strain specific differences in ISGylation have been found [15]. The IBV NS1 has an additional species preference for ISG15-conjugation, as only primate ISG15 is conjugated. Upon binding, IBV NS1 translocates ISG15 to the nucleus in species-dependent patterns [15], which is not observed with ISG15 from other species. We did not observe any clustering of neither s8ORF2 protein nor ISG15 in the nucleus of ISAV infected cells, suggesting that s8ORF2 does not inhibit ISGylation by mechanisms similar to that of IBV NS1. However, in the study of Svingerud *et al.* [20] infection with the highly virulent ISAV strain (Glesvaer), which was also used in the present study, induced lower levels of ISG15 compared to a low virulent ISAV strain 24 hpi. Future studies will determine whether there are strain differences in ISG15 conjugation of s8ORF2 protein. 

Interaction between the s8ORF2 protein and ubiquitin was also determined in the present study. How ubiquitin-like protein modulators orchestrate signaling-pathways are still not understood, and often less than 5% of their target protein pool are conjugated [14]. Nevertheless, for the small ubiquitin-like modifier SUMO it has been shown that transient modification of translational factors could have long-lasting downstream consequences through chromatin remodeling or recruitment of inhibitory complexes [48]. Apart from tagging proteins for degradation by the proteasome, ubiquitin is also involved in vesicle transport, signaling pathways and DNA-repair [49]. Together with ISG15 and SUMO they shape the strength and duration of innate immune responses [12]. In the present study, the ISAV s8ORF2 protein was shown to be conjugated to both ISG15 and ubiquitin. Further studies may reveal the molecular mechanisms involved in the ability of s8ORF2 protein to counteract the antiviral innate immune responses. 

An amino acid discrepancy was observed in position 154 in the s8ORF2 protein when comparing the MS and Genbank/in house sequencing data. The reason for this is not clear but spontaneous mutation in gene segment 8 during the final cultivation prior to the WB and MS analysis seems at present to be the most plausible explanation. A blastp search revealed that of the currently available ISAV segment 8 gene sequences covering this region (38), all but two contain a serine in this position. 

Since the s8ORF2 protein constitutes part of the virus particle, co-IP experiments 4 dpi were performed in order to determine whether the s8ORF2 protein was linked to other structural ISAV-proteins during virus particle assembly. We found that the s8ORF2 protein binds to NP, M and F protein, but not to NEP or HE. This might suggest that the s8ORF2 protein functions as a bridge between the viral ribonucleoprotein particles, and M and F, the latter extending through the outer lipid layer of the virus particle. The anti-NP antibody recognized several proteins smaller than that of the full-length NP protein (68 kDa), possibly representing cleavage products. The anti-s8ORF2 antiserum, on the other hand, produced bands with a higher MW than that predicted for the full-length protein (27.4 kDa), possibly representing modified forms of the protein. However, except for ISG15- and ubiquitinylation, we were not able to identify the type of modifications that altered the gel migration pattern. Moreover, both LC-MS analysis of proteins pulled down with anti-s8ORF2 antiserum and live imaging of cells expressing s8ORF2-EGFP suggest interactions with endogenous proteins. The cytosolic s8ORF2-EGFP protein did not diffuse freely out of the cell following plasma membrane permeabilization, as opposed to cells transfected with the EGFP vector where cytoplasmic staining diffused rapidly.

The proteins pulled down by anti-s8ORF2 antiserum strongly suggests that the s8ORF2 protein is involved in a multitude of steps important for ISAV replication, analogous to the versatile influenza virus NS1 protein. From the LC-MS analysis of putative interacting proteins, several proteins associated with cytoskeleton were pulled down, in addition to proteins linked to membrane rafts and mitochondrial membranes. 

Actin-myosin has been shown to be required for assembly of IAV [50]. Myosin-9, which is associated with actin and involved in transport and cell movements, was present in several of the excised protein bands. Proteins associated with actin- and intermediate filament binding and microtubules were also recorded. Virus assembly of budding enveloped viruses often occurs in lipid rafts regions of the cell membrane. Flotellin is one of the proteins involved in protein scaffolding into rafts [51]. Annexins, a class of Ca^2+^ and phospholipid binding proteins, are also involved in endocytosis and exocytosis. Annexin A5 has previously been found in influenza virus particles [52,53], and to be an important factor in counteracting the IFN-γ response and increase replication of the IAV both *in vitro* and *in vivo* [52]. The different annexins have different roles during endo- and exocytosis. In the present study annexin A1, A6 and A5 were pulled down. Molecular chaperones, regarded as general markers for stress responses and shown to be essential in the life cycle of several viruses [54,55,56], involved in translation, replication, transport and virus assembly were also identified. The heat shock protein 90 kDa (HSP90) and heat shock cognate 71 kDa protein (HSC71) were amongst the proteins pulled down. Furthermore, several ribosomal proteins and proteins involved in mRNA transport and -processing were also identified; the heterogeneous nuclear ribonucleoprotein M (hnRNR M), a nucleocytoplasmic shuttling RNA binding protein, has been shown to be a cleavage target by poliovirus and coxachievirus [57]; DDX5 has previously been shown to be important for replication of HIV-1 [58] and some other viruses [59,60]. Proteins like the mitochondrial inner membrane protein and voltage dependent anion selective channels (VDAC1 and 2) were also found present following IP. These proteins have important functions in transport across mitochondrial membranes and in apoptosis. VDAC-1 has previously been shown to be targeted by PB1-F2 of IAV to induce apoptosis [61]. No cytopathogenic effect was observed 4dpi in ISAV infected ASK cells. Later though, partial apoptotic cell death was observed, as well as alterations in mitochondrial morphology [62,63]. Galectin-9, a carbohydrate binding protein involved in immune response was also pulled down. This protein has been shown to be up-regulated during ISAV infection [64].

Since NP was present in several of the bands excised for LC-MS analysis there is also the possibility that some of the proteins detected are pulled down by NP, and hence are linked only indirectly to the s8ORF2 protein. However, most of the proteins pulled down are endogenous proteins known to play roles in the life cycle of several viruses. Further studies would be required in order to elucidate in more detail the function and type of interactions occurring between the s8ORF2 protein and host proteins, and to determine the specific significance these interactions have in the ISAV life cycle. 

## 5. Conclusions

In the present study, the functional properties of the type I IFN antagonistic RNA-binding protein encoded by ISAV s8ORF2 were investigated. We found that it shares several functional similarities with NS1 of IAV and IBV. Like these proteins the s8ORF2 protein has a N-terminal domain predicted to be responsible for RNA binding, is dimeric and capable of interfering with type I IFN-pathways, possibly by direct coupling to ISG15. However, in contrast to the influenza virus NS1 proteins, the s8ORF2 protein is present in the virus particle and mainly found in the cytosol. Together, this indicates that these multitasking proteins exert different roles during infection despite their common RNA-binding properties. Finally, a number of proteins, many well known to play central roles in life cycle of viruses were identified as possible interactors with the s8ORF2 protein. The present study provides new information on the functional properties of an ISAV protein involved in the establishment of the infection that may aid to the development of more efficient vaccines against ISA in the future.

## Figures and Tables

**Table 1:** LC/MS analysis of excised bands from SDS-PAGE.

Protein Band	^a^ Data-Base	Protein Name	NCBI Accession No.	Mw (kDa)	Spectrum Count	Function
1	*S. salar*	Myosin-9	224613261	60	13	Actin binding
	*S. salar*	Eukaryotic translation initiation factor 3 subunit	223647896	115	3	Translation
2	*S. salar*	Myosin-9	224613261	60	6	Actin binding
	*O. mykiss*	Actin beta	185132289	42	9	Actin
	*O. mykiss*	Heat shock 90 KDa protein 1 beta isoform	185132161	83	2	Chaperone
3	*S. salar*	Myosin-9	224613261	60	7	Actin binding
	*O. mykiss*	Heat shock 90 KDa protein 1 beta isoform	185132161	83	2	Chaperone
4	*O. mykiss*	Heat shock 90 KDa protein 1 beta isoform	185132161	83	16	Chaperone
	*S. salar*	Mitochondrial inner membrane protein	209153972	79	13	Transport
	*S. salar*	Myosin-9	224613261	60	11	Actin binding
	*S. salar*	Annexin A1	213510942	38	3	Multifunctional
	Virus	NP protein	313744891	68	2	ISAV
5	Virus	NP protein	313744891	68	14	ISAV
	*S. salar*	Myosin-9	224613261	60	12	Actin binding
	*S. salar*	Heat shock cognate 71 kDa protein	213514058	71	3	Chaperone
	*S. salar*	Myelin expression factor 2	291190830	65	3	Transcription
6	*S. salar*	Flotillin 1	213511228	47	7	Scaffolding protein
	*S. salar*	Myosin-9	224613261	60	5	Actin binding
	*S. salar*	Flotillin-2a	213514074	47	2	Scaffolding protein
	Virus	S8ORF2 protein	313754903	27	3	ISAV
	Virus	NP protein	313744891	68	2	ISAV
7	Virus	NP protein	313744891	68	70	ISAV
	*S. salar*	Myelin expression factor 2	291190830	65	8	Transcription
	*S. salar*	Heat shock cognate 71 kDa protein	213514058	71	7	Chaperone
	*S. salar*	Myosin-9	224613261	60	6	Actin binding
	*S. salar*	Heterogeneous nuclear ribonucleoprotein M	213512325	72	4	Pre-mRNA binding
	*S. salar*	Probable ATP-dependent RNA helicase DDX5	223649022	68	4	mRNA processing
	*S. salar*	Annexin A6	213514676	75	2	Multifunctional
	*S. salar*	Syncoilin	213512082	64	2	Intermediate filament
8	*S. salar*	Myosin-9	224613261	60	6	Actin binding
	*S. salar*	Galectin-9	209733430	38	4	Carbohydrate binding
	Virus	NP protein	313744891	68	3	ISAV
	*S. salar*	Annexin A1	213510942	38	3	Multifunctional
	*S. salar*	Capping protein (Actin filament) muscle Z-line α 2	213510872	33	3	Actin binding
	*S. salar*	F-actin-capping protein subunit-1	223647378	33	3	Actin binding
	Virus	S8ORF2 protein	313754903	27	2	ISAV
	*S. salar*	Voltage-dependent anion channel 3	213511881	30	2	Transport
9	Virus	S8ORF2 protein	313754903	27	13	ISAV
	*S. salar*	Capping protein (actin filament) muscle Z-line β	197631853	31	3	Actin binding
	Virus	NP protein	313744891	68	2	ISAV
	*O. mykiss*	Actin β	185132289	42	2	Actin
10	*S. salar*	Tropomyosin α-3 chain	218505649	29	16	Actin binding
	*O. mykiss*	Actin β	185132289	42	15	Actin
	*S. salar*	Tropomyosin α-3 chain	223647762	28	15	Actin binding
	*S. salar*	Tropomyosin α-4 chain	213515262	29	10	Actin binding
	*S. salar*	Voltage-dependent anion channel 2-2	197632613	30	8	Transport
	*S. salar*	40S ribosomal protein S3a	213514340	30	7	Ribosomal protein
	*S. salar*	Ribosomal protein S3-1	197632569	27	6	Ribosomal protein
	Virus	s8ORF2 protein	313754903	27	5	ISAV
	*S. salar*	Myosin-9	224613264	60	5	Actin binding
	*S. salar*	Ribosomal protein L7a	198285627	30	5	Ribosomal protein
	Virus	Tyrosine-protein kinase transforming protein Fgr	125357	62	4	Kinase, transferase
	*S. salar*	Voltage-dependent anion-selective channel protein 2	209154650	34	4	Transport
	*S. salar*	THO complex subunit 4	209733738	28	3	mRNA processing
	*S. salar*	60S ribosomal protein L6	213511212	30	2	Ribosomal protein
	*S. salar*	Annexin A5	213514536	35	2	Multifunctional
	*S. salar*	Capping protein (actin filament) muscle Z-line β	197631853	31	2	Actin binding
	*S. salar*	Voltage-dependent anion-selective channel protein 1	209156112	31	2	Transport

^a^ Databases are NCBI *Salmo salar*, NCBI *Oncorhynchus mykiss* and NCBI virus. Minimum two spectrum counts.

## Supplementary Materials

The following are available online at http://www.mdpi.com/1999-4915/8/2/52/s1, Figure S1: S8ORF2 protein is present in both the cytosol and nucleus of some ISAV infected ASK cells, Figure S2: Nucleo-cytoplasmic shuttling is not affected when s8ORF2-EGFP transfected cells are treated with Leptomycin B to inhibit CRM-1 dependent nuclear export, Figure S3: Western blot of co-immunopresipitation (IP) assays with s8ORF2 and F protein.
