# Supplementary material for: Infectious Salmon Anaemia Virus (ISAV) RNA Binding Protein Encoded by Segment 8 ORF2 and Its Interaction with ISAV and Intracellular Proteins
Source: Viruses. 2016 Feb 18;8(2):52. doi: 10.3390/v8020052 (PMC4776207; doi:10.3390/v8020052)
Supplement: Supplementary file 1 [file viruses-08-00052-s001.pdf]

Supplementary Materials: Infectious Salmon Anaemia Virus (ISAV) RNA Binding Protein Encoded by Segment 8 ORF2 and Its Interaction with ISAV and Intracellular Proteins

Christel M. Olsen [1], Turhan Markussen [1], Bernd Thiede [2] and Espen Rimstad [1,*]

Supplementary Materials: The following are available online at www.mdpi.com/link, Figure S1: S8ORF2 protein is present in both the cytosol and nucleus of some ISAV infected ASK cells, Figure S2: Nucleo-cytoplasmic shuttling is not affected when s8ORF2-EGFP transfected cells are treated with Leptomycin B to inhibit CRM-1 dependent nuclear export, Figure S3: Western blot of co-immunopresipitation (IP) assays with s8ORF2 and F protein.

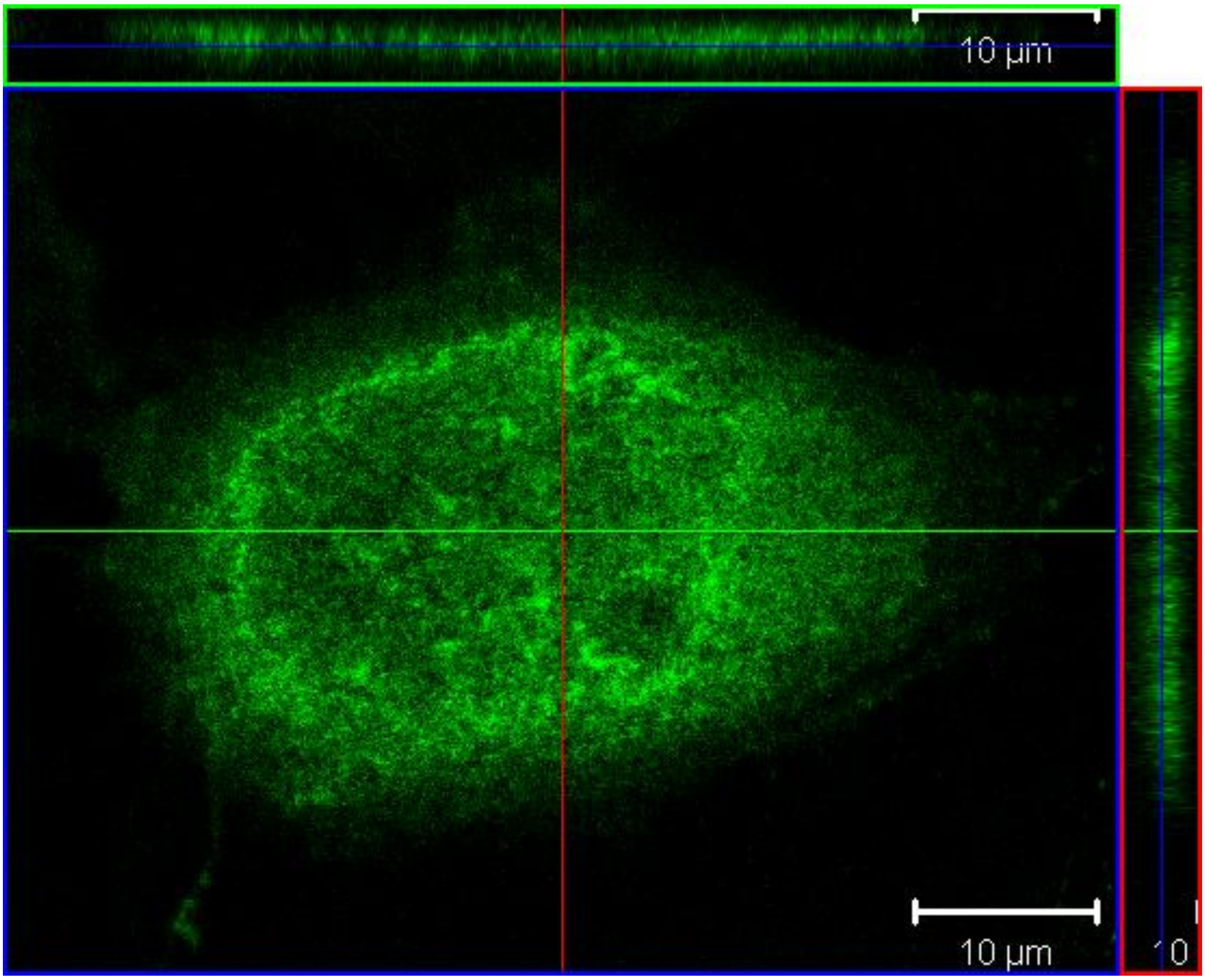

Figure S1. S8ORF2 protein is present in both the cytosol and nucleus of some ISAV infected ASK cells. Picture show x,y,z dimentions of confocal Z-stack of one ASK cell immunostained with anti-s8ORF2 antiserum 72 hpi. (Z = 3.71 µm). Picture was taken at 63× magnification in a confocal microscope.

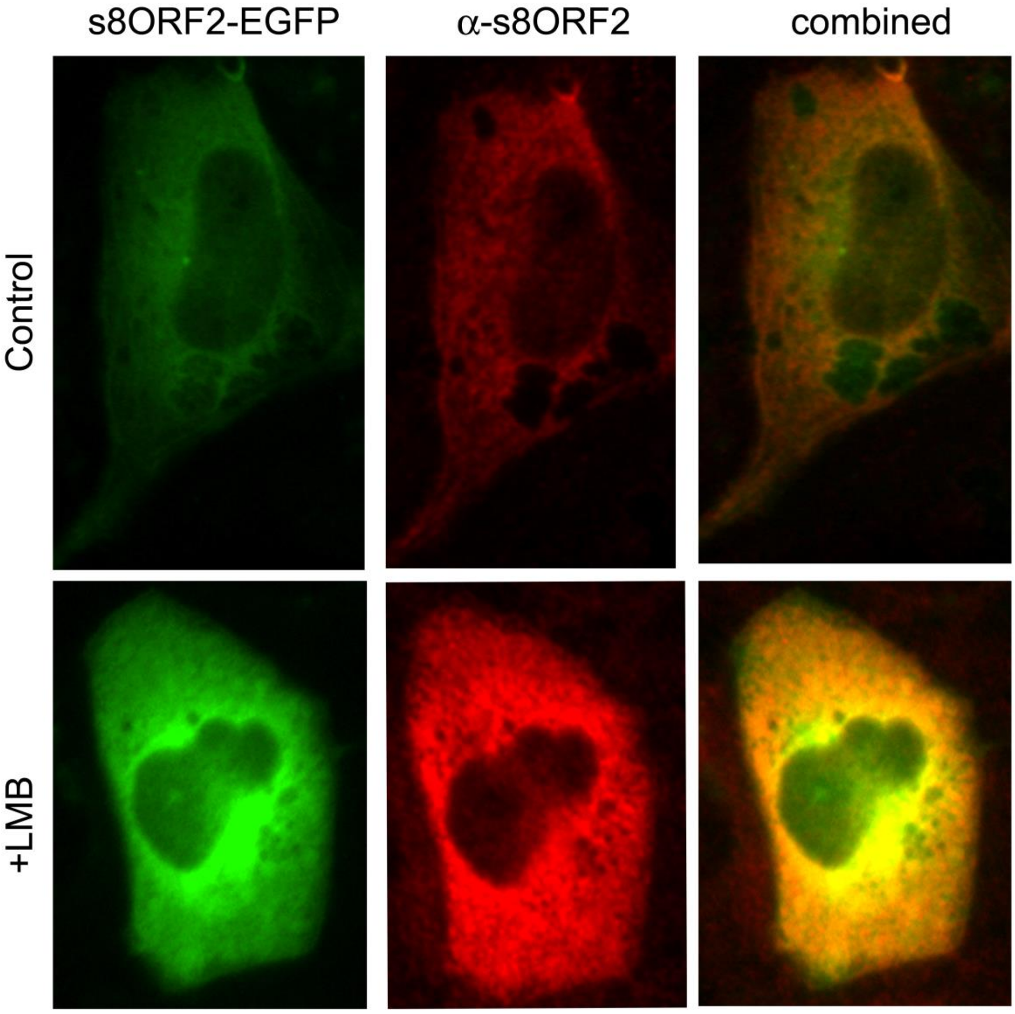

Figure S2. Nucleo-cytoplasmic shuttling of S8ORF2-EGFP protein is not affected when CRM-1 dependent nuclear export is inhibited by Leptomycin B. Chinook salmon embryo cells (CHSE-214) were transfected the same way as described for EPC cells with s8ORF2-EGFP (control) or treated for 4 h with 40 μM LMB before immunostaining with anti-s8ORF2 antiserum 24 h post transfection. Pictures were taken in inverted fluorescence microscope at 40× magnification and show EGFP (green), anti-s8ORF2 (red) and combined signals (yellow).

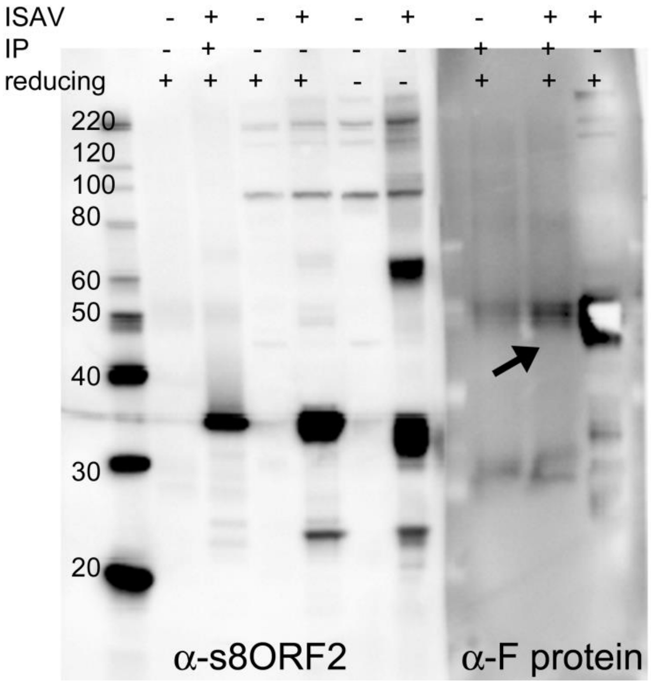

Figure S3. Western blots of co-immunoprecipitation (IP) assays with s8ORF2 and ISAV protein F. The anti-s8ORF2 blot is the same as in Figure 1, and the membrane developed by anti-F antiserum indicate that when using anti-s8ORF2 in pull-down this also pulls down proteins in the non-infected cell with same molecular weight as F-protein (arrow). Compared to the direct lysate (-IP) small amounts of F protein is pulled down with anti-s8ORF2 (arrow), however the interpretation of this result is disturbed by the non-specific signal with the same MW in IP from non-infected cells.

© 2016 by the authors; licensee MDPI, Basel, Switzerland. This article is an open access article distributed under the terms and conditions of the Creative Commons by Attribution (CC-BY) license (http://creativecommons.org/licenses/by/4.0/).
